# Supplementary material for: Impact of High-Dose Methotrexate on the Outcome of Patients with Diffuse Large B-Cell Lymphoma and Skeletal Involvement
Source: Cancers (Basel). 2021 Jun 12;13(12):2945. doi: 10.3390/cancers13122945 (PMC8231126; doi:10.3390/cancers13122945)

Table S1. Chemotherapy regimens.

Abbreviation	Chemotherapy Regimen	Dose	Days
**ACBVP**	Doxorubicin	75 mg/m2	d1
	Cyclophosphamide	1200 mg/m2	d1
	Vindesine	2 mg/ m2/d	d1-d5
	Bleomycin	10 mg/m2/d	d1-d5
	Prednisone	60 mg/m2/d	d1d5
	Carmustine	300 mg/m2	d1
**BEAM**	Cytarabine	200 à 400 mg/m2/d	d2d5
	Etoposide	200 à 400 mg/m2/d	d2d5
	Melphalan	140 mg/m2	d6
**CHOP**	Doxorubicin	50 mg/m2	d1
	Cyclophosphamide	750 mg/m2	d1
	Vincristine	1,4 mg/m2	d1
	Prednisone	40 mg/m2	d1d5
**COPADEM**	Adriamycin	60 mg/m2	d2
	Methotrexate	3 g/m2	d1
	Vincristine	1,4 mg/m2	d1
	Cyclophosphamide	500 mg/m2	d2d4
	Dexamethasone	40 mg	d1d4
**DHAP**	Cisplatin	100 mg/m2	d1
	Cytarabine	2 g/m2 x 2	d2
	Dexamethasone	40 mg/j	d1d4
**ESAP**	Cisplatin	25 mg/m2	d1d4
	Etoposide	40 mg/m2/d	d1d4
	Cytarabine	2 g/m2/d	d5
	Methylprednisone	500 mg/d	d1d5
**MBACOD**	Methotrexate	3 g/m2	d8
	Bleomycin	10 mg/m2	d1
	Cyclophosphamide	600 mg/m2	d1
	Doxorubicin	45 mg/m2	d1
	Vincristine	1 mg/m2	d1
	Dexamethasone	6 mg/m2	d1d5

Table S2. Characteristics of patients who received radiotherapy or not.

**Characteristic**	**Whole Cohort (n=93)**	**No Radiotherapy (n=53)**	**Radiotherapy (n=40)**
**Age**	57 (48 - 68)	58 (48 - 69)	57 (48 - 75)
**Age > 60 years**	42 (45%)	25 (47%)	17 (43%)
**Gender (M/F)**	52/41	27/26	25/15
**ECOG > 1**	46 (50%)	28 (53%)	18 (45%)
**High LDH serum levels**	71 (76%)	43 (81%)	28 (70%)
**Stage III/IV**	80 (86%)	48 (91%)	32 (80%)
**aaIPI**			
**0 - 1**	19 (20%)	7 (13%)	12 (30%)
**2 - 3**	74 (80%)	46 (87%)	28 (70%)
**CNS_IPI**			
0 - 1	13 (14%)	4 (7%)	9 (23%)
2 - 3	33 (35%)	21 (40%)	12 (30%)
≥ 4	47 (51%)	28 (53%)	19 (47%)
**Sites of Skeletal Involvement**			
**Localized PBL**	13 (14%)	5 (9%)	8 (20%)
**Multifocal PBL**	17 (18%)	9 (17%)	8 (20%)
**Secondary Bone Lymphoma**	63 (68%)	39 (74%)	24 (60%)
**Sites of Skeletal Involvement**			
**Axial**	84 (90%)	51 (96%)	33 (83%)
**Skull**	7 (8%)	7 (13%)	0
**Vertebral**	58 (62%)	33 (62%)	25 (63%)
**Rib Cage**	23 (25%)	16 (30%)	7 (18%)
**Pelvis**	42 (45%)	27 (51%)	15 (38%)
**Distal**	36 (39%)	20 (38%)	16 (40%)
**Lower Limb**	23 (25%)	12 (23%)	11 (28%)
**Upper Limb**	21 (23%)	12 (23%)	9 (23%)
**Epidural Involvement**	33 (36%)	16 (30%)	17 (43%)
**Bone Marrow Involvement**	36 (39%)	22 (42%)	14 (35%)
**Prior Low-Grade Lymphoma**	14 (15%)	9 (17%)	5 (13%)
**Treatment**			
**Initial Chemotherapy Regimen**			
**CHOP**	79 (85%)	50 (94%)	29 (73%)
**MBACOD**	12 (13%)	2 (4%)	10 (25%)
**Other**	2 (2%)	1 (2%)	1 (3%)
**High-Dose Methotrexate**	50 (54%)	24 (45%)	26 (65%)
**Platinum-based Consolidation**	21 (23%)	9 (17%)	12 (30%)
**High-Dose Cytarabine Consolidation**	37 (40%)	17 (32%)	20 (50%)
**Intra-Thecal Therapy**	39 (42%)	25 (47%)	14 (35%)
**Autologous SCT**	39 (42%)	21 (40%)	18 (45%)

aaIPI, age-adjusted International Prognostic Index; CNS-IPI, Central Nervous System-IPI; ECOG, Eastern Cooperative Oncology Group; LDH: Lactate DeHydrogenase; PBL, Primary Bone Lymphoma ; SCT, Stem Cell Transplantation.

Table S3. Characteristics of the whole cohort and of patients who received autologous stem cell transplantation or not.

Characteristic	Landmark cohort (n=84)	No ASCT (n=46)	ASCT (n=38)
**Age**	57 (46 - 65)	63 (50 - 72)	51 (43 - 57)
**Age > 60 years**	34 (41%)	29 (63%)	5 (13%)
**Gender (M/F)**	48/38	24/22	24/14
**ECOG > 1**	37 (44%)	19 (41%)	18 (47%)
**High LDH serum levels**	62 (74%)	33 (72%)	29 (76%)
**Stage III/IV**	72 (86%)	35 (76%)	37 (97%)
**aaIPI**			
**0 - 1**	19 (23%)	14 (30%)	5 (13%)
**2 - 3**	65 (77%)	32 (70%)	33 (87%)
**Type of Lymphoma**			
**Localized PBL**	12 (14%)	11 (24%)	1 (3%)
**Multifocal PBL**	17 (20%)	8 (17%)	9 (24%)
**Secondary Bone Lymphoma**	55 (66%)	27 (59%)	28 (74%)
**Sites of Skeletal Involvement**			
**Axial**	75 (89%)	38 (83%)	37 (97%)
**Skull**	6 (7%)	2 (4%)	4 (11%)
**Vertebral**	51 (61%)	24 (52%)	27 (71%)
**Rib Cage**	21 (25%)	10 (22%)	11 (29%)
**Pelvis**	39 (46%)	19 (41%)	20 (53%)
**Distal**	33 (39%)	20 (44%)	13 (34%)
**Lower Limb**	20 (24%)	10 (22%)	10 (26%)
**Upper Limb**	20 (24%)	14 (30%)	6 (16%)
**Epidural Involvement**	31 (37%)	12 (26%)	19 (50%)
**Bone Marrow Invovelment**	32 (38%)	12 (26%)	20 (53%)
**Prior Low-Grade Lymphoma**	13 (16%)	2 (4%)	11 (29%)
**Treatment**			
**Initial Chemotherapy Regimen**			
**CHOP**	70 (83%)	41 (89%)	29 (76%)
**MBACOD**	12 (14%)	4 (9%)	8 (21%)
**Other**	2 (2%)	1 (2%)	1 (3%)
**High-Dose Methotrexate**	50 (60%)	17 (37%)	33 (87%)
**Platinum-based Consolidation**	19 (23%)	5 (11%)	14 (37%)
**High-Dose Cytarabine Consolidation**	35 (42%)	8 (17%)	27 (71%)
**Intra-Thecal Therapy**	37 (44%)	18 (39%)	19 (50%)
**Radiotherapy**	39 (46%)	21 (46%)	18 (47%)

aaIPI, age-adjusted International Prognostic Index; ECOG, Eastern Cooperative Oncology Group; LDH: Lactate DeHydrogenase; PBL, Primary Bone Lymphoma ; SCT, Stem Cell Transplantation.

Fig S1. Progression-free survival (PFS), in patients < 60 years (A) and > 60 years (B), and overall survival (OS), in patients < 60 years (C) and > 60 years (D), treated with or without HD-MTX.

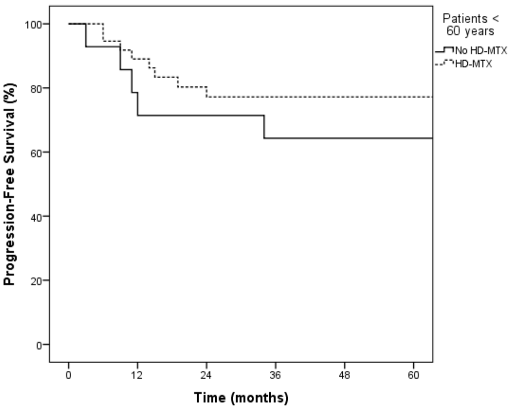

A

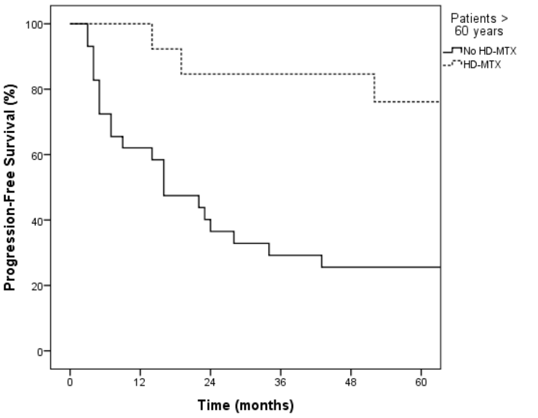

B

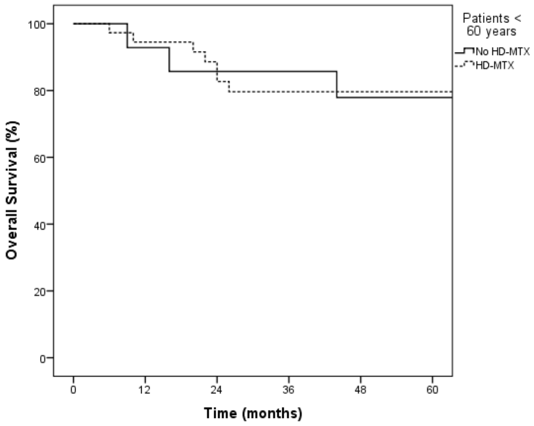

C

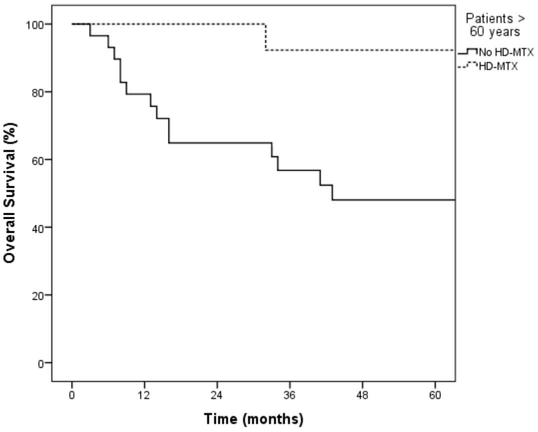

D

Fig S2. Progression-free survival (PFS), in patients with a low or intermediate CNS-IPI (A) and a high CNS-IPI (B), and overall survival (OS), in patients with a low or intermediate CNS-IPI (C) and a high CNS-IPI (D), treated with or without HD-MTX.

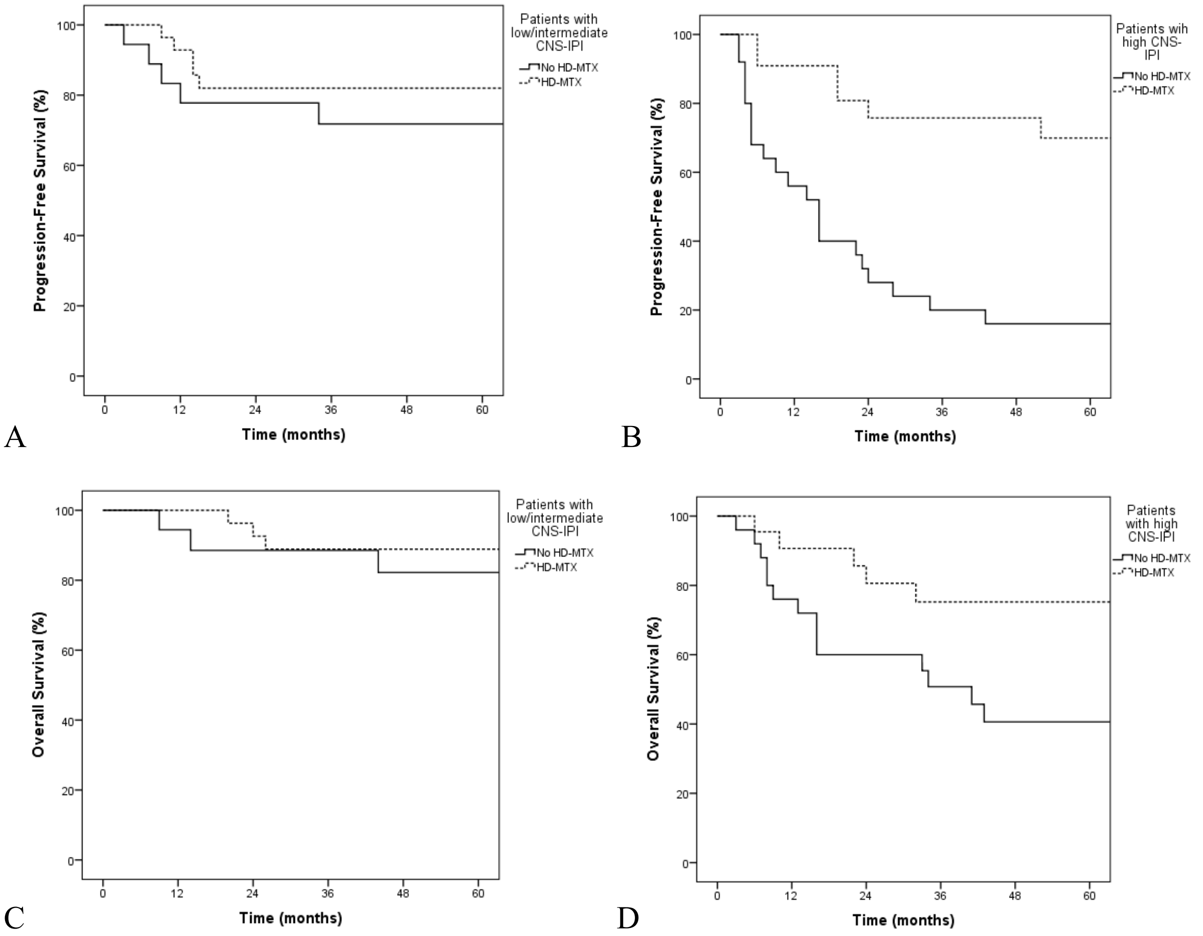

Fig S3. Progression-free survival (PFS) (A) and overall survival (OS) (B) of patients with secondary bone lymphoma treated with or without HD-MTX.

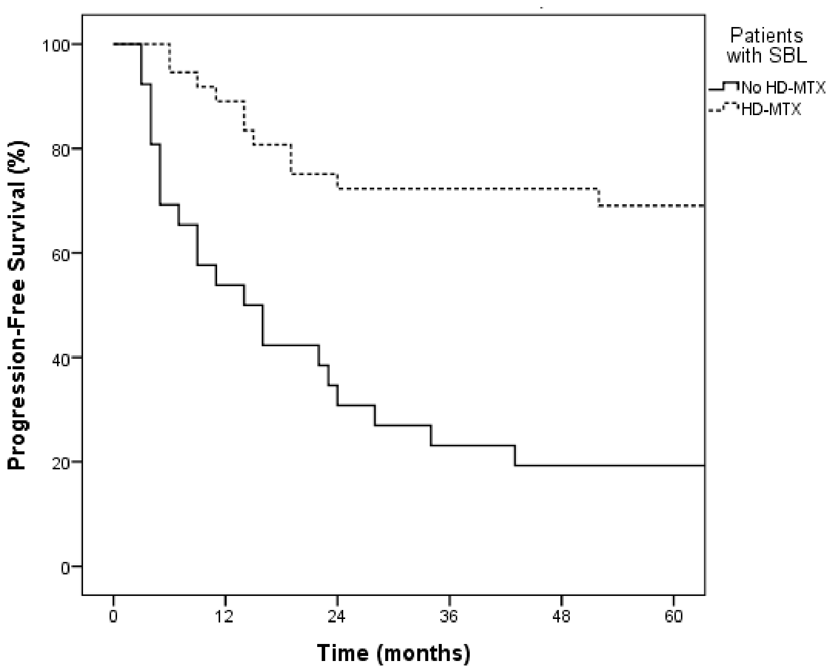

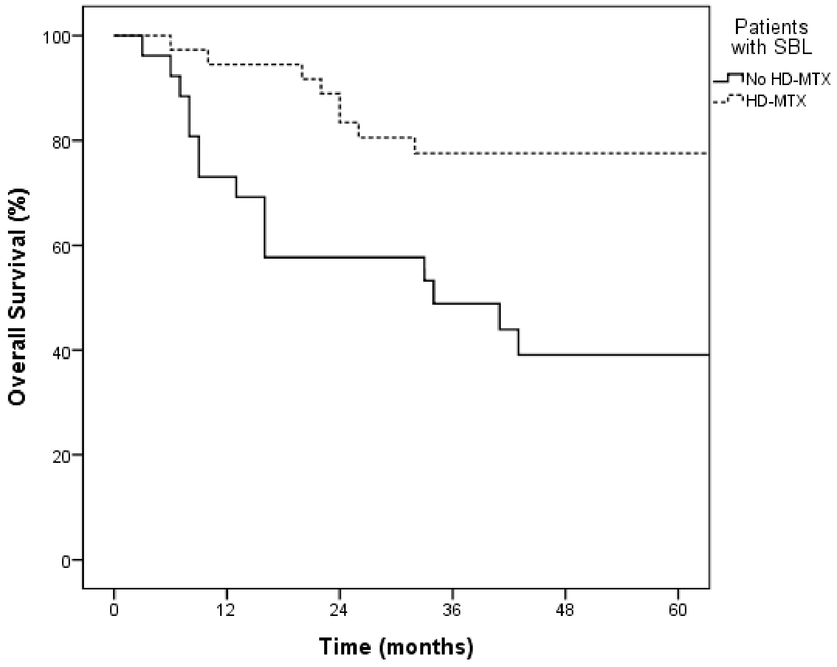

Supplement: Supplementary file 1 [file cancers-13-02945-s001.zip › cancers-1246024-supplementary.pdf]
